# Targeted Metabolomics Revealed a Sex-Dependent Signature for Metabolic Syndrome in the Mexican Population

**DOI:** 10.3390/nu14183678

**Published:** 2022-09-06

**Authors:** Berenice Palacios-González, Guadalupe León-Reyes, Berenice Rivera-Paredez, Isabel Ibarra-González, Marcela Vela-Amieva, Yvonne N. Flores, Samuel Canizales-Quinteros, Jorge Salmerón, Rafael Velázquez-Cruz

**Affiliations:** 1 Scientific Bonding Unit, Medicine Faculty UNAM-INMEGEN, Mexico City 14610, Mexico; 2 Genomics of Bone Metabolism Laboratory, National Institute of Genomic Medicine (INMEGEN), Mexico City 14610, Mexico; 3 Research Center in Policies, Population and Health, School of Medicine, National Autonomous University of Mexico (UNAM), Mexico City 04510, Mexico; 4 Institute of Biomedical Research, IIB-UNAM, Mexico City 04510, Mexico; 5 Laboratory of Inborn Errors of Metabolism, National Pediatrics Institute (INP), Mexico City 04530, Mexico; 6 Epidemiological and Health Services Research Unit, Morelos Mexican Institute of Social Security, Cuernavaca 62000, Mexico; 7 Department of Health Policy and Management and UCLA-Kaiser Permanente Center for Health Equity, Fielding School of Public Health, University of California, Los Angeles, CA 90095, USA; 8 UCLA Center for Cancer Prevention and Control Research, Fielding School of Public Health and Jonsson Comprehensive Cancer Center, Los Angeles, CA 90095, USA; 9 Unit of Genomics of Population Applied to Health, Faculty of Chemistry, National Autonomous University of Mexico (UNAM), Mexico City 04510, Mexico; 10 National Institute of Genomic Medicine (INMEGEN), Mexico City 14610, Mexico

**Keywords:** sexual dimorphism, metabolism, acylcarnitines, amino acid, uric acid

## Abstract

Metabolic syndrome (MetS) is a group of several metabolic conditions predisposing to chronic diseases. Individuals diagnosed with MetS are physiologically heterogeneous, with significant sex-specific differences. Therefore, we aimed to investigate the potential sex-specific serum modifications of amino acids and acylcarnitines (ACs) and their relationship with MetS in the Mexican population. This study included 602 participants from the Health Workers Cohort Study. Forty serum metabolites were analyzed using a targeted metabolomics approach. Multivariate regression models were used to test associations of clinical and biochemical parameters with metabolomic profiles. Our findings showed a serum amino acid signature (citrulline and glycine) and medium-chain ACs (AC14:1, AC10, and AC18:10H) associated with MetS. Glycine and AC10 were specific metabolites representative of discrimination according to sex-dependent MetS. In addition, we found that glycine and short-chain ACs (AC2, AC3, and AC8:1) are associated with age-dependent MetS. We also reported a significant correlation between body fat and metabolites associated with sex-age-dependent MetS. In conclusion, the metabolic profile varies by MetS status, and these differences are sex-age-dependent in the Mexican population.

## 1. Introduction

Metabolic syndrome (MetS) is a group of several metabolic conditions that predisposes to chronic diseases such as osteoarthritis and specific gastrointestinal cancers, including colon cancer and hepato-cellular carcinoma [1,2,3]. The prevalence of MetS is increasing worldwide and it varies between 21% and 38% around the word [4]. The Mexican National Nutrition Survey (ENSANUT), according to the Adult Treatment Panel III (ATP III) criteria, reported that the prevalence of MetS in Mexican adults is >50% and rises with age in a sex-specific manner [5]. Women are more frequently affected than men due to the higher central obesity, physiological alterations, and hormonal changes during and after menopause [6]. MetS risk factors include age, heredity, lifestyle habits, and low physical activity [7]. Heritability studies have estimated from 13–30% for MetS and more than 50% for some specific metabolic components [8]. 

In chronic metabolic disorders like MetS, the phenotype is complex and dynamic because of several interactions among heritable and environmental factors. In this context, metabolomics is a powerful phenotyping tool. It offers metabolic profiles representing an integrated view of metabolism because it allows sensitive detection of molecular modifications over time, resulting from the interaction between intrinsic and extrinsic causes. Also, metabolomics provides a comprehensive signature or biomarkers to reveal early metabolic dysfunctions [9]. Individuals diagnosed with MetS are physiologically heterogeneous because of the complex nature of the syndrome, inducing discrepancies in the diagnosis and therapeutic approach [10].

Furthermore, significant clinical differences between men and women with MetS have been reported [11,12]. According to previous reports, the human metabolism is turned sex-specific; for example, women store more adipose tissue and have higher insulin sensitivity than men, while men tend to oxidize more lipids than women [13]. In healthy French volunteers, higher concentrations of creatinine, branched-chain amino acids (BCAAs), and small-chain acylcarnitines (ACs) were observed in men versus women [14]. In addition, it has been observed that the metabolism of BCAAs could be impacted not only by sex but also by ethnicity and gene expression [15,16,17].

In this context, studies with a metabolomic approach have tried to uncover insights into the pathology behind obesity, T2D, cardiovascular disease, and MetS [18,19,20]. It has been suggested that BCAAs are involved in insulin resistance [21]; together with aromatic amino acids (AAAs), BCAAs could aid as biomarkers to predict the start of MetS [22]. In addition, histidine and lysine have been shown to have antioxidant properties, decreasing the inflammatory burden and oxidative stress in MetS [23,24]. Conversely, alterations of circulating ACs are related to metabolic disorders [25,26]. Some reports have found that ACs attenuate MetS by modulation of tissue fatty acids [27] and decreasing inflammatory factors [28]. On the contrary, it has also been suggested that ACs may be involved in metabolic dysfunction, increasing fasting triglycerides and glucose and reducing insulin sensitivity [29]. 

Currently, there are no studies that have explored the possible changes in the serum metabolites and their association with MetS in a sex-specific manner in the Mexican population. Therefore, we aimed to investigate the potential sex-specific serum modifications of amino acids and ACs and their relationship with MetS in the Mexican population. This approach could become a powerful method for identifying individuals in subclinical stages, with a high risk of disease, besides identifying biomarker molecules related to the MetS disease mechanisms.

## 2. Materials and Methods

### 2.1. Health Workers Cohort Study (HWCS)

This is a prospective open-label cohort study with follow-up investigations averaging six years and including participants enrolled between 2004 and 2018. The design and details of the study have been published previously [30]. In summary, the study aimed to analyze the influence of genetic and lifestyle factors on the occurrence of diverse chronic diseases. The blood samples and data were collected from health workers in various professions and their relatives from standard care clinics in Cuernavaca, Morelos. For this analysis, 602 subjects aged ≥18 with blood samples who had participated in the third wave between 2016 and 2018 were selected. The protocol was approved by IMSS (No. 12CEI 09 006 14, 17 May 2016) and the National Institute of Genomic Medicine (346–05/2018/I, 5 August 2018), following the Declaration of Helsinki (13/LO/0078). All participants provided written informed consent. Age was categorized as <45 and ≥45 years old because, based on previous studies, there are endocrine changes affecting metabolic traits in women and men around 45 years [31,32,33].

### 2.2. Metabolic Syndrome Criteria

MetS and its components were evaluated according to the ATP III criteria. The diagnosis of MetS was established by the presence of three or more of the following five criteria: (1) abdominal obesity (waist circumference [WC] ≥ 102 cm for men or ≥88  cm for women); (2) triglycerides ≥ 150 mg/dL and/or drug treatment for elevated triglycerides; (3) high-density lipoprotein cholesterol (HDL-c) < 40 mg/dL for men, and <50 mg/dL for women; (4) systolic blood pressure (BP) ≥ 130 or diastolic BP > 85 mmHg or antihypertensive medication treatment and/or a history of hypertension; and (5) fasting glucose ≥ 100 mg/dL, and/or treatment with medications for T2D [34].

### 2.3. Covariates

Height, weight, WC, and BP were measured using standardized procedures (reproducibility was evaluated, resulting in a concordance coefficient of 0.83–0.90). Body weight was measured with a calibrated electronic scale (model BC-533; Tanita), height using a conventional stadiometer (SECA), and WC with a standard steel measuring tape (SECA brand). BP was measured after 5 minutes of rest in a sitting position with an electronic digital BP monitor. Two BP measurements were obtained and the mean was used. Body composition (lean mass and fat mass) was measured by dual-energy X-ray absorptiometry using a Lunar DPX NT instrument (Lunar Radiation Corp.) following standard procedures. All DXA scans were performed by certified and trained technicians, who positioned the participants in a supine position with their hands level with their hips and feet slightly apart. Daily quality controls were performed according to the guidelines provided by the manufacturer.

### 2.4. Other Measurements

Serum samples for measuring glucose, transaminases (alanine aminotransferase [ALT] and aspartate aminotransferase [AST]), total cholesterol [TC], HDL-c, and triglyceride levels were obtained after overnight fasting. The clinical measurements were determined using commercial tests that have been previously reported [30]. All biomedical assays were performed at the IMSS laboratory in Cuernavaca with procedures standardized according to the proceedings of the International Federation of Clinical Chemistry and Laboratory Medicine [35]. Demographic characteristics and detailed past medical history were obtained using a self-administered questionnaire. 

### 2.5. Targeted Metabolomics Analysis

Concentrations of forty serum metabolites (ACs, free carnitine (AC0), and amino acids) were measured using the approach of targeted metabolomics as previously described [36]. NeoBase MSMS kit is intended for the quantitative determination of ACs: Free carnitine (AC0), Acetyl acylcarnitine (AC2), Propionyl acylcarnitine (AC3), Butyryl acylcarnitine (AC4), Isovaleryl acylcarnitine (AC5), Tiglyl acylcarnitine (AC5:1), Hexanoyl acylcarnitine (AC6), Adipoyl acylcarnitine (AC6DC), Octanoyl acylcarnitine (AC8), Octenoyl acylcarnitine (AC8:1), Hexadecanoyl acylcarnitine (AC16), Hexadecenoyl acylcarnitine (AC16:1), Hydroxy Hexadecenoyl acylcarnitine (AC16:1OH), Hydroxy Hexadecanoyl acylcarnitine (AC16OH), Decanoyl acylcarnitine (AC10), Decenoyl acylcarnitine (AC10:1), Decadienoyl acylcarnitine (AC10:2), Dodecanoyl acylcarnitine (AC12), Dodecenoyl acylcarnitine (AC12:1), Tetradecanoyl acylcarnitine (AC14), Tetradecenoyl acylcarnitine (AC14:1), Tetradecadienoyl acylcarnitine (AC14:2), Hydroxy tetradecenoyl acylcarnitine (AC14OH), Octadecanoyl acylcarnitine (AC18), Octadecadienal acylcarnitine (AC18:1), Hydroxy octadecenoyl (AC18:1OH), Octadecadienal acylcarnitine (AC18:2), and Hydroxy octadecanoyl (AC18OH); amino acids: Glycine, Alanine, Valine, Leucine, Methionine, Phenylalanine, Tyrosine, Ornithine, Citrulline, Arginine, and Proline; and the ketone Succinylacetone. Briefly, 20 μL of serum were poured onto filter paper cards (Whatman 903, Dassel, Germany) and dried at room temperature. The spot was cut into 2-mm circles and placed in a 96-well plate. The extraction solution was added to the plate and incubated for 30 min at 30 °C at 650× *g*. Finally, 10 μL of each sample was injected into the flow at 4-min intervals. The Micromass Quattro equipment (Waters Inc., Milford, MA, USA) was coupled with an ESI source in positive mode. Nitrogen gas was used for desolvation and nebulization, and argon was the collision gas.

### 2.6. Statistical Analyses

Sociodemographic and clinical characteristics of individuals by sex and age groups were compared using Wilcoxon test and Pearson’s Chi-square test for continuous and categorical variables, respectively. All datasets were normalized. The selected methods were Row-wise normalization: Normalization to constant sum; Data transformation: Log10 Normalization; and Data scaling: Mean Centering. We used partial least-squares discriminant analysis (PLS-DA) to identify discrimination among samples. Permutation testing was conducted to minimize the possibility that the observed separation on PLS-DA was by chance. Additionally, to the cross-validation, a model validation was performed by a 2000 times permutation test. A loading scatter plot was constructed to determine the variables discriminating between the groups. Variable importance in projection (VIP) plot was performed for ranking the metabolites based on their significance in discerning studies from both groups. VIP cutoff > 1.0 was designated since the number of variables in this study was less than 100. Unsupervised hierarchical clustering heatmaps were performed. The 25 top metabolites obtained by ANOVA analysis were selected to be shown. All statistical analyses were performed using MetaboAnalyst 5.0 (McGill University, Toronto, ON, Canada) [37]. 

## 3. Results

### 3.1. Demographics and Clinical Characteristics of the Study Population 

**Figure 1:**
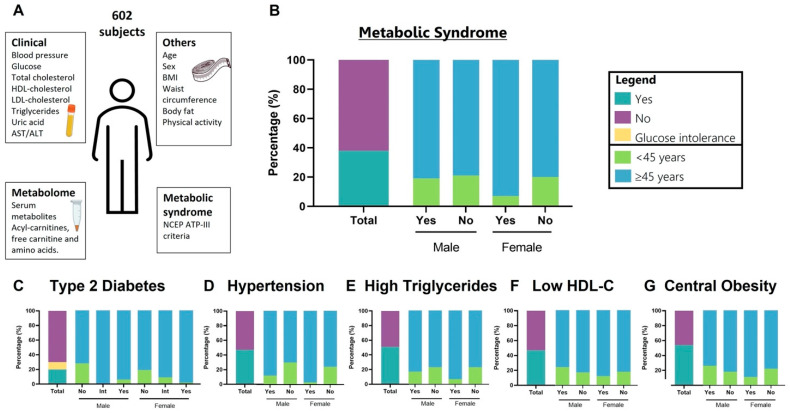
Clinical characteristics of the 602 individuals belonging to the Health Workers Cohort. (**A**) Summary of MetS parameters measured. Each outlined square indicates a different data type and schematically describes the different measurements. (**B**) Percentage of individuals with MetS according to the NCEP-ATP III criteria. Left: global percentage, purple: subjects with the condition, dark green: subjects without the disease. Right: subdivided into males and females. Light green: <45 years subjects, blue: ≥45 years subjects. (**C**–**G**), percentage of individuals with each of the five criteria used for MetS diagnosis is displayed in a similar style to (**B**): yellow designates subjects with glucose intolerance.

Baseline characteristics of 602 participants are summarized in Figure 1. In the overall population, the median age was 60 years (range 50–68), and 75.9% were females. The prevalence of overweight and obesity was 66%. Thirty-seven percent met the criteria for MetS, according to the ATP III criteria. The prevalence of MetS-ATP III was higher in women than men (40.3% vs. 29.7%, *p* = 0.022). Both men and women with MetS were older and had a higher prevalence of obesity, BP, glucose, triglycerides, and liver transaminases than those without MetS (Table 1).

### 3.2. Serum Metabolite Profile According to Metabolic Syndrome

**Figure 2:**
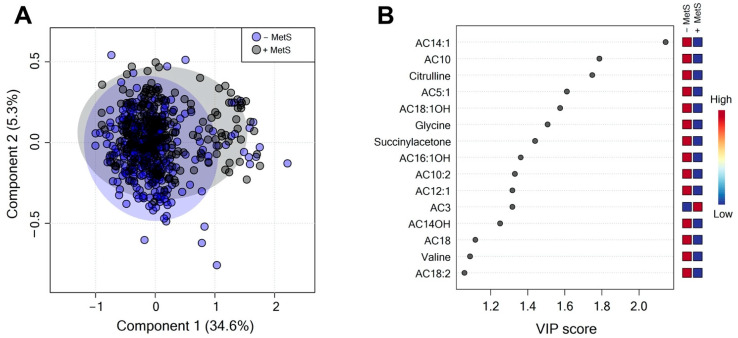
Serum metabolic profile according to MetS status. (**A**) PLS-DA plot shows differences between groups; −MetS (gray circles), +MetS (purple circles). The explained variances are shown in brackets (accuracy 0.63; R2 0.096; Q2 0.027: permutation *p* value < 5.0). (**B**) VIP analysis represents the relative contribution of metabolites to the variance among groups. A high VIP score represent a greater influence of the metabolites to the group separation. Red and blue boxes on the right, indicate whether metabolite concentration is increased (red) or decreased (blue). −Mets (without MetS), +MetS (with MetS).

**Figure A1:**
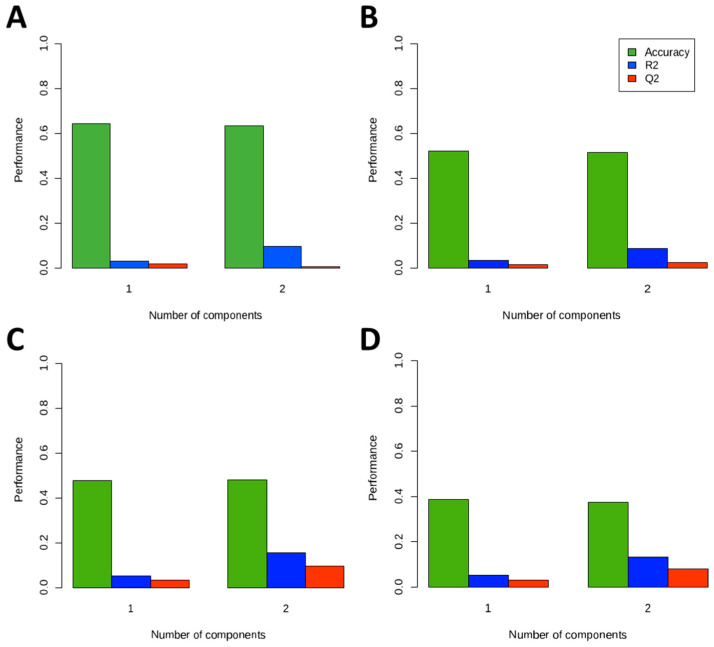
The explained variances. (**A**) MetS status (accuracy 0.63; R2 0.096; Q2 0.027); (**B**) MetS status and age (accuracy 0.51; R2 0.086; Q2 0.040); (**C**) MetS status and sex (accuracy 0.48; R2 0.155; Q2 0.086); (**D**) MetS, sex, and age (accuracy 0.375; R2 0.133; Q2 0.081).

A total of 40 serum metabolites were quantified. The PLS-DA score plots showed slight evidence of separation according to MetS status (Figure 2A). Despite this slight difference between groups (accuracy 0.63; R2 0.096; Q2 0.027: permutation *p* value < 5.0) (Appendix A; Figure A1), the VIP plot revealed that AC14:1, AC10, citrulline, AC5:1, AC18:1OH, glycine, and succinyl acetone are responsible for discrimination between the groups, which made them potentially useful for discrimination (Figure 2B). These metabolites are decreased in the +MetS group (Figure 2B).

### 3.3. Metabolic Profile According to Sex-Dependent MetS 

**Figure 3:**
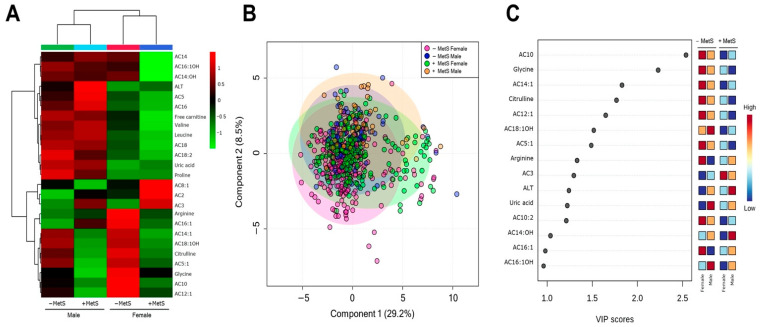
Serum metabolite profile according to MetS status and sex. (**A**) Hierarchical heatmap for MetS diagnosis and sex: Males: −MetS (green) and +MetS (light blue); Females: −MetS (red) and +MetS (dark blue). Red and green show increasing and decreasing concentration, respectively. (**B**) PLS-DA plot shows separation between Males −MetS (blue), Males +MetS (yellow), Females −MetS (pink), and Females +MetS (green). The explained variances are shown in brackets (accuracy 0.48; R2 0.155; Q2 0.086: permutation *p* value < 5.0). (**C**) VIP analysis represents the relative contribution of metabolites to the variance among groups. A high VIP score indicates a greater contribution of the metabolites to the group separation**.** Red and blue boxes on the right indicate whether metabolite concentration is increased (red) or decreased (blue). −Mets (without MetS), +MetS (with MetS).

It has been reported that there are significant clinical differences between males and females with MetS. An unsupervised hierarchical clustering of abundance heatmap revealed differences by sex and among the MetS groups (Figure 3A). Interestingly, AC3 concentration was MetS-dependent and independent of sex. Arginine and AC16:1 were higher only in females without MetS; meanwhile, AC8:1 was higher only in females with MetS. The PLS-DA score plots showed slight evidence of separation according to MetS and sex (Figure 3B). The explained variances are shown in brackets (accuracy 0.48; R2 0.155; Q2 0.086: permutation *p* value < 5.0) (Appendix A; Figure A1). The VIP plot showed that AC10 (higher in −MetS groups, regardless of sex) and glycine (higher in −MetS groups, regardless of sex) are helpful for discrimination between groups (Figure 3C).

### 3.4. Metabolic Profile According to Age-Dependent MetS 

**Figure 4:**
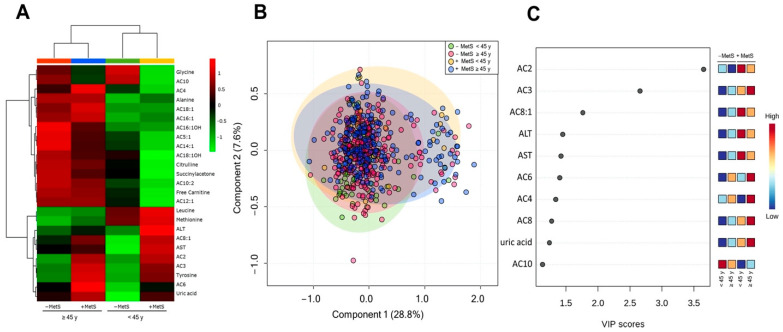
Serum metabolite profile according to MetS status and age. (**A**) Hierarchical heatmap for MetS diagnosis and age: −MetS < 45 years (green), −MetS ≥ 45 years (red), +MetS < 45 years (yellow), and +MetS ≥ 45 years (blue), red and green show an increase and decrease concentration, respectively. (**B**) PLS-DA plot shows separation between groups: −MetS: <45 years (green) and ≥45 years (red); +MetS: <45 years (yellow) and ≥45 years (blue). The explained variances are shown in brackets (accuracy 0.51; R2 0.086; Q2 0.040: permutation *p* value < 5.0). (**C**) VIP analysis represents the relative contribution of metabolites to the variance among groups. A high VIP score indicates a greater contribution of the metabolites to the group separation. Red and blue boxes on the right indicate whether metabolite concentration is increased (red) or decreased (blue). −Mets (without MetS), +MetS (with MetS).

As mentioned above, metabolic disorders are age-dependent. An unsupervised hierarchical clustering of abundance heatmap showed differences according to age and MetS status (Figure 4A). Glycine and AC10 concentrations were age-independent and MetS-dependent. Leucine and Methionine concentrations were higher in younger subjects independent of MetS presence. The PLS-DA score plots revealed slight evidence of separation according to MetS and age (Figure 4B). The explained variances are shown in brackets (accuracy 0.51; R2 0.086; Q2 0.040: permutation *p* value < 5.0) (Appendix A; Figure A1). The VIP plot showed that AC2, AC3, and AC8:1 are useful for discrimination between groups (Figure 4C). The +MetS groups showed higher concentration of AC2, AC3, and AC8:1 (Figure 3C).

### 3.5. Metabolic Profile According to Sex-Age Dependent MetS 

**Figure 5:**
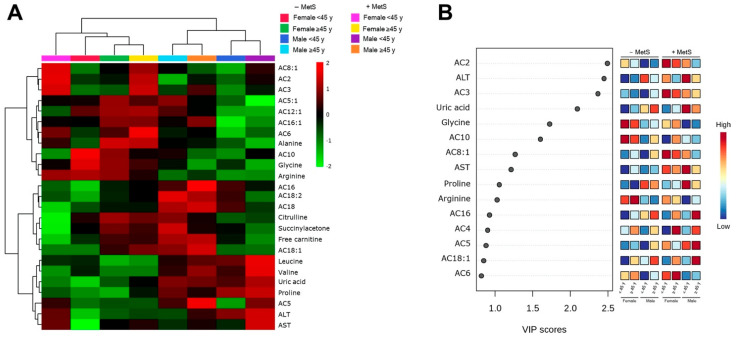
Serum metabolite profile according to MetS, sex, and age. (**A**) Hierarchical heatmap for MetS diagnosis, sex, and age: Females: −MetS: <45 years (red) and ≥45 years (green); +MetS: <45 years (pink) and ≥45 years (yellow). Males: −MetS: <45 years (dark blue) and ≥45 years (light blue); +MetS: <45 years (purple) and ≥45 years (orange). The explained variances are shown in brackets (accuracy 0.375; R2 0.133; Q2 0.081: permutation *p* value < 5.0). (**B**) VIP analysis represents the relative contribution of metabolites to the variance among groups. A high VIP score indicates a greater contribution of the metabolites to the group separation. Red and blue boxes indicate whether metabolite concentration is increased (red) or decreased (blue). −Mets (without MetS), +MetS (with MetS).

An unsupervised hierarchical clustering of abundance heat map showed a separation between groups by sex and age (Figure 5A). The male cluster was age-associated regardless of MetS diagnosis. Interestingly, both men and women ≥ 45 years +MetS show a similar pattern in AC5 concentrations and liver enzymes (ALT and AST) (Figure 5A). Furthermore, we observed that women with MetS, regardless of age, have the same ACs pattern (AC2, AC3, and AC8:1). For BCAAs (Leucine and Valine), uric acid (UA), and proline, we observed a gender-dependent cluster independent of MetS diagnosis (Figure 5A). The explained variances are shown in brackets (accuracy 0.375; R2 0.133; Q2 0.081: permutation *p* value < 5.0) (Appendix A; Figure A1). The VIP plot showed AC2 (higher in women with MetS, regardless of age, and younger men with MetS diagnosis), ALT (higher in men with MetS, regardless of age, and younger women with MetS diagnosis), AC3 (higher in MetS groups, regardless of sex), UA (higher in men with MetS diagnosis regardless of age), and glycine (higher in women −MetS group, regardless of age) are suitable for discrimination between groups (Figure 5B).

**Figure A2:**
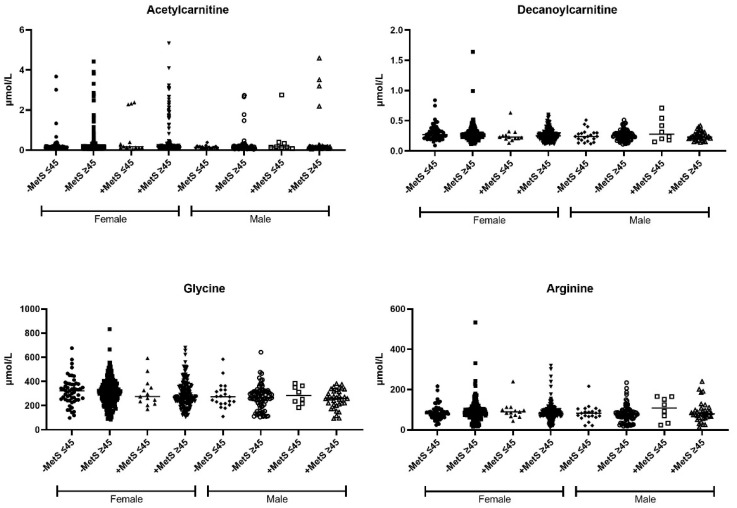
Serum concentration of acetylcarnitine, decanoylcarnitine, glycine, and arginine according to MetS, sex, and age.

After adjustment for sex and age, metabolites such as glycine, AC10, arginine, and AC2 remained significant (Table 2; Appendix A: Table A1 and Figure A2).

### 3.6. Correlation between Body Fat and Metabolites Associated with Sex-Age Dependent MetS

Previous studies have reported that as body fat mass increases, abdominal fat mass and percentage of whole-body fat mass are related to MetS components such as BP, dyslipidemia, obesity, and T2D [38,39,40]. Correlations between body fat mass and metabolites associated with sex, age, and MetS were performed (Table 3). In healthy women (−MetS) and aged women with MetS, arginine and glycine were negatively correlated with body fat mass. The AC10 levels in younger (<45 years) healthy (−MetS) women and aged men with MetS significantly correlates with body fat mass (−0.36 and 0.40, respectively). 

## 4. Discussion

In the present study, a targeted metabolomic approach was applied to characterize the sex-age-specific serum amino acids and ACs and their relationship with MetS in the Mexican population. Timely identification of MetS physiological disruptions should allow pinpointing individuals at the highest risk of developing T2D, cardiovascular disease, and multi-organ damage. In addition, understanding the factors contributing to these sex and age differences may help reduce disparities in outcomes observed across age- and sex-based subgroups. A complete understanding of the metabolic changes across age- and sex-based subgroups are needed to improve our mechanistic and clinical knowledge of such differences.

ACs are derived from mitochondrial and peroxisomal Acyl-CoA metabolites by substituting the carnitine moiety for CoA. They are synthesized by the enzyme carnitine palmitoyltransferase 1 (CPT 1). The general role of ACs is to transport fatty acids into the mitochondrial matrix to produce energy. This process is known as β-oxidation [41,42]. Incomplete fatty acid oxidation results in elevated ACs concentrations [43], which is used in newborn screening to detect metabolic disorders [44]. Higher concentrations of ACs in individuals with obesity and T2D have been associated with incomplete fatty acid oxidation (FAO). The proposed mechanism is related to an increase in β-oxidation; it causes an accumulation of acetyl-CoA, which exceeds the tricarboxylic acid (TCA) cycle rate, leading to an incomplete β-oxidation [45,46]. Also, higher ACs concentrations cause imbalances between the synthesis and secretion of insulin, which consequently causes β-cell dysfunction [47]. 

AC2 is classified as a short-chain AC. This is produced by the mitochondrial matrix enzyme, CrAT, from carnitine and acetyl-CoA, a molecule that is a product of both fatty acid β-oxidation and glucose oxidation and can be used by the acid TCA for energy generation [48]. Alterations in AC2 levels were already reported in prediabetic states [49]. Pujos-Guillot et al. suggest ACs levels could be used to predict MetS [39]. Also, high levels of ACs are associated with fatty liver and cardiovascular disease [50,51]. In our study, men, and women under 45 years of age without and with MetS, show the highest concentrations of AC2 compared to the other groups. This finding could indicate a risk for the future development of MetS in the case of men who have not yet developed MetS. Future longitudinal studies will be necessary to corroborate this hypothesis.

AC10 is classified as a medium chain AC. These are formed either through esterification with L-carnitine or through the peroxisomal metabolism of longer chain-AC [52]. The Carnitine octanoyltransferase enzyme (CrOT) is responsible for the synthesis of all medium-chain AC (AC5–AC12) in peroxisomes [53]. Many medium-chain ACs can serve as useful markers for inherited disorders of fatty acid metabolism. In particular, AC10 is elevated in the blood or plasma of individuals with obesity in adolescence [54]. 

In our study, AC10 was associated with body fat percentage negatively in healthy younger women and positively in aged men with MetS. Our results agree with other studies on male adults with obesity [49,50,55]. During obesity, incomplete oxidation of FAO results in a broadscale increase in plasma ACs intermediates (short and medium-chain ACs) [56,57]. In contrast, in this study, younger women with lower body fat percentages showed higher concentrations of AC10. Previous studies have found similar results in children and adolescents [57]. The researchers propose that, unlike adults with impaired FAO characterized by obesity and insulin resistance, obese adolescents with T2D have lower concentrations of ACs intermediates and higher rates of β-oxidation. This finding is attributed to the chronicity of obesity and the consequent and gradual evolution of failure of mitochondrial adaptive mechanisms as the obese individual transitions from youth to adulthood and forward with continued obesity [56,57]. It is important to point out that the presence of greater adiposity in young women without a diagnosis of MetS shows higher concentrations of AC10, which could indicate a risk for the future development of obesity or insulin resistance. 

However, Sunny et al. indicated that insulin stimulation resulted in higher oxidation rates of BCAAs, contributing to higher levels of AC5 in plasma [58]. The increased concentrations of BCAAs overload the catabolic pathways in the liver and skeletal muscle, increasing the production of succinyl-CoA and propionyl-CoA, reducing the β-oxidation of fatty acids and the catabolism of glucose. Therefore, the loss of efficiency in oxidative metabolic pathways amplifies the oxidation of partially oxidized products, increasing mitochondrial stress, reducing insulin sensitivity, and altering circulating glucose concentrations [59,60]. In the present study, men under 45 years diagnosed with MetS have the highest concentrations of BCAAs and proline. Our findings agree with recent studies reporting impaired BCAAs metabolism in many metabolic diseases associated with obesity, insulin resistance, and T2D [61,62,63]. 

On the other hand, when comparing subjects of both sexes older than 45 years with MetS, it was observed that women have lower concentrations of BCAAs and proline. Recent studies found that males had significantly higher BCAAs, AAAs, proline, and ornithine [15]. The authors suggest that the differences observed in BCAAs levels show sex-related heterogeneity in BCAA catabolism [64]. Regarding proline concentrations, several authors indicate that a high proline profile is associated with obesity, insulin resistance, hypertriglyceridemia, and decreased glucose-stimulated insulin secretion [22]. Our results agree with the literature; when we separated the gender groups and their insulin sensitivity, the women who presented lower sensitivity to the hormone regardless of the presence of MetS showed higher concentrations of proline (Appendix A: Table A1). In addition, chronic exposure to proline in β-cells has been associated with impairment in insulin gene transcription and mitochondrial oxidative phosphorylation [65]. 

UA is associated with the risk of developing MetS, and it has even been proposed that it could be used as an additional marker in MetS [51,66,67]. Regarding UA concentrations in our study, younger men diagnosed with MetS had the highest UA concentrations. Unlike men, regardless of age and MetS diagnosis, women had lower UA concentrations. Our results agree with many studies that point to clear sex-specific disparities regarding the UA concentration, owing to various factors [68]. Potential explanations for sex differences in the UA level include genetic background for urate production and renal and intestinal excretion, certain bacteria residing in the gut that can metabolize one-third of the daily urate load produced endogenously, and the exogenous urate from dietary purines [69]. Recently, an enrichment of the enzyme’s glycine dehydrogenase subunit and the glycine reductase complex were observed in the intestinal microbiota of patients with gout. The authors propose that the gut microbiome of gout patients not only contributes to nucleotide salvage and de novo purine biosynthesis pathways but also performs vital functions in the de novo synthesis of purine precursors (such as glycine) [69].

In healthy individuals, glycine is biosynthesized in the body from the amino acid serine. Serine is mostly derived from the diet, but it can also be produced from glucose via 3-phosphoglycerate, especially in kidneys. In addition to being synthesized from serine, glycine can also be derived from threonine, choline, or hydroxyproline via inter-organ metabolism of the liver and kidneys [70].

Glycine is a precursor for porphyrins, purines, creatine, and sarcosine, it inhibits protein glycation, it increases hepatic pyruvate production, and it plays an essential role in DNA methylation, intracellular redox balance, and bile acid conjugation [71]. In addition, lower glycine concentration is associated with hepatic steatosis and can predict impaired glucose tolerance, dyslipidemia, and T2D [72,73,74]. In the present study, after adjusting for age and gender, lower glycine levels were significantly associated with MetS, ALT, AST, and T2D diagnosis. Decreased blood glycine and serine levels have previously been shown in adult patients with asymptomatic hyperuricemia or gout compared with healthy adult controls. Furthermore, in clinical and population studies, ALT has been shown to predict T2D and MetS, given its relationship with insulin resistance and central obesity. This result supports the hypothesis that liver injury can be induced by metabolically active intra-abdominal fat [40]. Moreover, serum glycine concentration has been considered the only homeostatic assessment–insulin resistance (HOMA-IR)-associated predictor of adiposity in functionally limited overweight elders [75]. 

On the other hand, arginine is an essential amino acid. Infants are unable to effectively synthesize arginine, making it nutritionally essential for infants. However, in adults the major TCA cycle intermediate α-ketoglutarate is at the heart of generating multiple amino acids. In particular, the interconversion of glutamate to α-ketoglutarate allows the generation of several amino acids, including arginine. In addition, Ornithine aminotransferase (OAT) generates ornithine, which can generate arginine through the urea cycle [76].

This amino acid is involved in the synthesis of various products responsible for regulatory functions in the body. In particular, nitric oxide (NO) regulates carbohydrate and lipid metabolism [77]. The arginine is involved in multiple NO-dependent pathways that favor the whole-body oxidation of fatty acids and glucose [78], enhancing insulin sensitivity. In addition, recent studies have demonstrated that arginine activates the mammalian target of rapamycin (mTOR) cell signaling pathway in skeletal muscle to enhance protein synthesis and whole-body growth [70]. Therefore, the preceding could indicate that those individuals with the highest body fat percentage would present lower muscle mass, which could have lower insulin sensitivity. Interestingly, glycine and arginine negatively correlate with body fat percentage in healthy women regardless of age (without MetS) and aged women with MetS. However, this correlation was not observed in males and younger females with MetS; this could be attributed to changes in hormonal secretion and loss of estrogen in menopause, which causes an accumulation of visceral fat, leading to abdominal obesity [79,80]. Abdominal obesity is responsible for insulin resistance and the development of MetS [81]. Another possible explanation for the observed discrepancy can be attributed to sampling size, particularly in men with MetS.

This study identified a sex-dependent metabolomic signature associated with MetS. It is essential to mention that current treatments do not consider sex as a biological variable; yet, as we observed, there are apparent differences in metabolites. Therefore, these findings suggest starting to consider treatments with a sex-dependent approach. However, replication studies and knowledge of normal levels of individual metabolites in serum are necessary before translating these results into clinical practice.

This study has some limitations. First, the participants are a select group of health workers recruited from the Central region of Mexico (Cuernavaca, Morelos), and this may not reflect the health behavior of the entire Mexican population; therefore, additional studies are necessary before the observed findings can be generalized to individuals from other areas of Mexico. Second, the sample size of the men included in the work could limit the analysis to detect metabolites with minor effects on MetS and sex-specific effects. Third, although we analyzed the diet of individuals (one of the main determinants of human blood metabolites), we did not observe a dietary pattern associated with the metabolite profile, possibly due to insufficient sample size. Fourth, our cohort is very heterogeneous. Future studies need to consider the stratification of the population with common characteristics to minimize inter-individual variations and improve the predictive value. The present study has several strengths. First, to our knowledge, this is the first targeted metabolomics study on MetS in the Mexican population. The population study involved in this study includes men and women, which raises the consistency of our results. Second, some of the metabolites (e.g., glycine and AC10) identified in this analysis have been previously related to serum glucose levels and central obesity, components of MetS. This reference supports the confidence of our findings and could represent a good and exciting metabolite set related to MetS. Further studies, including a larger group of men and a broader panel of metabolites, are needed to confirm our results and clarify possible molecular pathways for associations detected between identified serum metabolites, fat mass, and MetS.

## 5. Conclusions

In summary, we used a targeted metabolomic analysis to identify plasma metabolic profiles in MetS patients. We found two ACs (AC2 and AC10) and two amino acids (glycine and arginine) that showed good potential to distinguish MetS patients from healthy individuals. We found in this study that ACs and amino acids are biomarkers that possibly provide a risk prediction and a novel tool for monitoring disease progression. Given the increase in the prevalence of MetS observed in the Mexican population, studies investigating the incidence of MetS and its characteristics from the point of view of omics technologies will be essential to unravel the pathophysiological mechanisms underlying the disease. Further studies should focus on evaluating the role of body fat in early metabolic health, insulin resistance, UA levels, and their role in the development of MetS. 

## Figures and Tables

**Table 1:** Baseline characteristics of the study population.

	Overall	Female			Male		
Characteristics		−MetS	+MetS		−MetS	+MetS	
	*n* = 602	*n* = 273	*n* = 184	*p* value	*n* = 102	*n* = 43	*p* value
Age, (years) ^a^	60 (50–68)	58 (49–65)	63 (57–71)	<0.001	55 (46–63)	59 (49–67)	0.365
BMI, (kg/m^2^) ^a^	26.0 (24.1–30.5)	25.3 (22.9–28.3)	29.5 (26.8–33.4)	<0.001	25.4 (23.3–27.6)	30.1 (27.2–32.4)	<0.001
Overweight, %	39	37.7	39.1	0.762	45.1	32.6	0.162
Obesity, %	27.2	16.1	46.2	<0.001	11.8	53.5	<0.001
WC, (cm) ^a^	93 (86–100)	86 (81–94)	98 (92–104)	<0.001	94 (89–99)	106 (99–109)	<0.001
Body fat (%)	42.8 (36.1–47.8)	43.4 (38.9–47.7)	47.2 (43.0–50.8)	<0.001	30.9 (28.1–33.8)	35.1 (33.1–38.3)	0.0001
DBP, (mmHg) ^a^	75 (69–82)	74 (67–79)	76 (69–83)	0.0009	77 (71–82)	85 (79–91)	<0.001
SBP, (mmHg) ^a^	120 (109–134)	114 (105–123)	127 (116–142)	<0.001	120 (111–132)	132 (122–145)	<0.001
Fasting plasma glucose, (mg/dL) ^a^	99 (92–109)	96 (89–102)	105 (95–120)	<0.001	98 (93–107)	108 (95–119)	0.015
Total cholesterol, (mg/dL) ^a^	198 (169–224)	198 (172–225)	199 (172–235)	0.666	194 (162–222)	199 (162–220)	0.843
HDL-c, (mg/dL) ^a^	50.7 (42.3–59.8)	55.7 (49.7–65.4)	47.7 (41.1–56.2)	<0.001	46.7 (40.8–54)	39.5 (34.7–48)	0.001
LDL-c, (mg/dL) ^a^	113.1 (90.9–135.8)	115.1 (93.3–134.9)	110.1 (88.1–139)	0.416	118.3 (90.3–138.9)	102.9 (87.3–131.7)	0.101
Triglycerides, (mg/dL) ^a^	141 (105–197)	123 (94–150)	181 (139–226)	<0.001	129 (99–175)	196 (166–282)	<0.001
Uric acid, (mg/dL) ^a^	5.2 (4.4–6.2)	4.7 (4.1–5.5)	5.4 (4.6–6.4)	<0.001	5.8 (5.2–6.8)	6.7 (5.7–7.8)	0.010
AST, (U/I) ^a^	25 (21–32)	24 (21–30)	27 (23–35)	0.002	26 (22–32)	30 (23–43)	0.057
ALT, (U/I) ^a^	26 (19–37)	29 (24–37)	35 (24–65)	0.004	29 (24–37)	35 (24–65)	0.016

^a^ Median (P25–P75). Abbreviations: Alanine aminotransferase (ALT); Aspartate aminotransferase (AST); Body Mass Index (BMI); Diastolic blood pressure (DBP); High-density Lipoprotein-cholesterol (HDL-c); Low-density Lipoprotein-cholesterol (LDL-c); Systolic blood pressure (SBP); Waist circumference (WC). *U* Mann-Whitney test was used. *p* < 0.05 was considered statistically significant.

**Table 2:** Linear models with covariate adjustments.

	LogFC	Abundance	t	*p* Value	Adj. *p* Value	β
Glycine	−0.027	−8.04 × 10^−18^	−4.19	3.19 × 10^−5^	0.0004	1.128
AC10	−0.019	−3.84 × 10^−18^	−3.57	0.0003	0.0024	−1.191
Arginine	−0.018	−3.53 × 10^−18^	−2.92	0.0036	0.0156	−3.283
AC2	0.031	−6.29 × 10^−18^	2.44	0.0146	0.0477	−4.541
AC16:1	−0.008	2.99 × 10^−18^	−2.03	0.0421	0.1084	−5.453

**Table 3:** Pearson correlation between body fat mass and metabolites associated with MetS.

		Females				Males			
		−MetS		+MetS		−MetS		+MetS	
		<45 years	≥45 years	<45 years	≥45 years	<45 years	≥45 years	<45 years	≥45 years
Arginine	r	−0.50	−0.26	0.38	−0.31	0.32	0.10	−0.68	0.27
	95% CI	−0.67 to −0.27	−0.38 to −0.13	−0.24 to 0.78	−0.44 to −0.16	−0.16 to 0.67	−0.12 to 0.31	−0.96 to 0.28	−0.11 to 0.57
	*p* value	0.0001	0.0001	0.219	<0.0001	0.186	0.386	0.134	0.163
Glycine	r	−0.66	−0.16	0.45	−0.17	0.30	0.05	−0.42	0.25
	95% CI	−0.79 to −0.49	−0.29 to −0.03	−0.16 to 0.81	−0.31 to −0.01	−0.17 to 0.66	−0.16 to 0.27	−0.92 to 0.58	−0.12 to 0.56
	*p* value	<0.0001	0.014	0.136	0.031	0.200	0.614	0.396	0.186
AC10	r	−0.36	−0.06	0.35	−0.06	0.16	0.06	−0.72	0.40
	95% CI	−0.57 to −0.11	−0.20 to 0.06	−0.27 to 0.77	−0.22 to 0.09	−0.31 to 0.57	−0.15 to 0.28	−0.96 to 0.21	0.05 to 0.67
	*p* value	0.005	0.331	0.255	0.407	0.506	0.565	0.106	0.027

## Data Availability

The datasets analyzed in this study are available from the corresponding author on reasonable request.

## Appendix A

### Proline Levels and Glucose Tolerance

Women with lower glucose tolerance have significantly higher concentrations of proline. In the men group, regardless of the presence of MetS, no significant changes were observed in proline concentrations relating to glucose tolerance.

**Table A1:** Proline concentration according to glucose tolerance.

	<45 Years		≥45 Years		
	No	Intolerance	No	Intolerance	T2D
Males	256.4 ± 18.48	294.6 ± 27.40	210.7 ± 8.60	237.7 ± 13.76	238.3 ± 11.58
Females	197.8 ± 60.51	239 ± 63.48 *	200.5 ± 64.21 ^b^	205.3 ± 61.75 ^b^	223.2 ± 59.02 ^a^

* Females < 45 years: None vs. Intolerance; Different letters indicate significant differences among groups (Females ≥ 45 years). *p* ≤ 0.05.
